# ﻿*Parathlasia* gen. nov. (Hemiptera, Cicadellidae, Ledrinae, Ledrini), a new leafhopper genus from Guizhou, China

**DOI:** 10.3897/zookeys.1138.82224

**Published:** 2023-01-06

**Authors:** Yu-Jian Li, Li-Na Jiang, Zi-Zhong Li, Ji-Chun Xing

**Affiliations:** 1 School of Life Sciences, Qufu Normal University, Qufu, Shandong Province 273165, Qufu, China Qufu Normal University Qufu China; 2 Institute of Entomology, Guizhou University, Guiyang, Guizhou Province 550025, Guiyang, China Guizhou University Guiyang China

**Keywords:** Auchenorrhyncha, Homoptera, key, *Midoria*, morphology, new genus, new species, taxonomy, *Thlasia*, *Yelahanka*

## Abstract

*Parathlasia***gen. nov.**, a new leafhopper genus and species of Ledrini, *P.guizhouensis***sp. nov.**, from Guizhou, China are described. Morphological differences between the new genus to other related Chinese genera are discussed. A key to distinguish *Parathlasia* from other similar genera is given.

## ﻿Introduction

**Figure 1.**
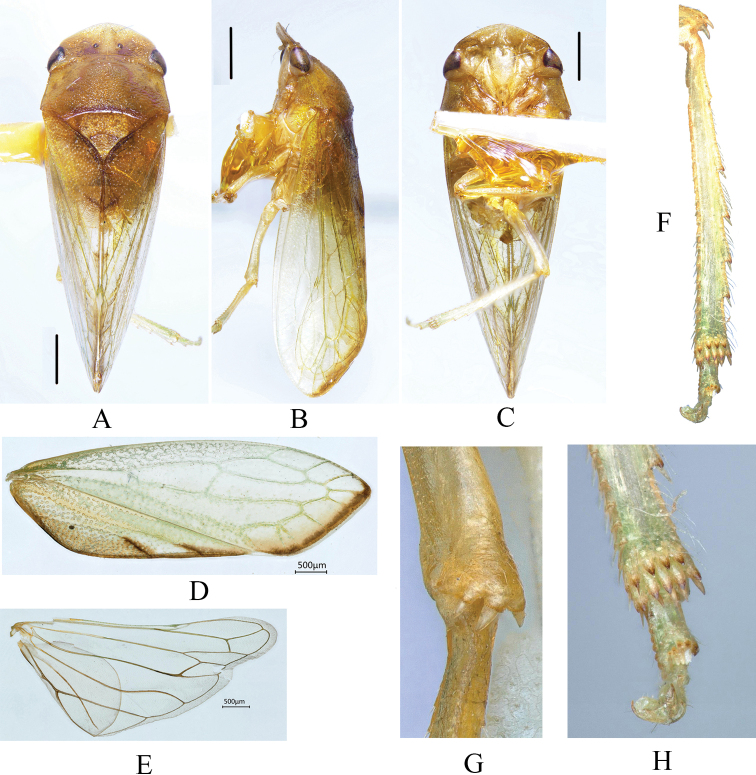
External morphology of *Parathlasiaguizhouensis* sp. nov. **A** dorsal habitus **B** lateral habitus **C** ventral habitus **D** forewing **E** hindwing **F** hind tibia **G** apex of hind femora **H** apex of hind leg.

The leafhopper subfamily Ledrinae is a rather special group with many prominent and unique features (Jones and Deitz 2009). It is a large group distributed worldwide with a preference for the tropics and subtropics, usually feeding on trees and shrubs. Of the four (Dietrich 2005) or five (Jones and Deitz 2009) recognized tribes the largest, Ledrini, comprises leafhoppers with a dorsum coarsely pitted or knobbed, lamellate or foliaceous anterolaterally with the head spatulate and face generally concave (Fig. 1A, B), forewings punctate with extra apical veins (Fig. 1D) or venation reticulate in the apical two-thirds. China is one of the main distribution areas of Ledrinae in the world (Li and Li 2008), with more than 160 species belonging to 23 genera. While sorting and identifying ongoing samples of ledrine leafhoppers from China, we found a new genus (with one new species) similar in appearance to *Thlasia* Germar but sharing similarities also with other Chinese genera, which are extensively described and illustrated below.

## ﻿Material and methods

Terminology used in this study is mainly based on Dietrich (2005) and Jones and Deitz (2009). Dry specimens were used for preparing descriptions and illustrations. External morphology was observed under a stereoscopic microscope. Body length was measured with an ocular micrometer, in millimeters, from the apex of head to the apex of the forewing at rest. Genital segments were examined and macerated in 10% KOH solution, washed in water and transferred to glycerin. Illustrations were made by eye using a Leica MZ 12.5 stereomicroscope. Multiple photographs were taken with a Leica D-lux 3 digital camera. Final digital images were compiled into Adobe Photoshop for labeling and plate composition. Specimens studied are deposited at the School of Life Sciences, Qufu Normal University, Qufu, China (**QFNU**) and the Institute of Entomology, Guizhou University, Guiyang, China (**GUGC**).

### ﻿Key to known genera of Ledrinae from China

**Table:** 

1	Tibia of hindfoot flat, foliaceous	**2**
–	Tibia of hindfoot, not foliaceous	**9**
2	Pronotum usually prominent, humped-like; with lateral extensions	**3**
–	Pronotum often declivous or weakly prominent; without extensions but lateral area concave	**4**
3	Lateral edge of pronotum laminately subangularly dilated	***Eleazara* Distant**
–	Lateral edge of pronotum straight	***Complanledra* Cai & He**
4	Lateral area of pronotum with ear shaped protrusions or longitudinal ridges	***Ledra* Fabricius**
–	Lateral area of pronotum without ear shaped protrusions	**5**
5	Crown elongate, middle length of crown greater than width between eyes	***Ledropsis* White**
–	Crown not significantly elongated, middle length of crown less than width between eyes	**6**
6	Crown ridge present	**7**
–	Crown without or weak ridge	***Confucius* Distant**
7	Forewing without a developed, sclerotized tubercle at first split of M vein	***Paraconfucius* Cai**
–	Forewing with a developed, sclerotized tubercle at first split of M vein	**8**
8	Crown with 2 window-like patches; bend at end of style with a small protuberance	***Funkikonia* Kato**
–	Crown without window-like patch; bend at end of style without a small protuberance	***Kuohledra* Cai & He**
9	Forewing cells strongly depressed, forewing veins raised	***Dusuna* Distant**
–	Forewing cells not strongly depressed, forewing veins raised not significantly	**10**
10	Lateral edge of pronotum protrude in an angular shape	**11**
–	Lateral edge of pronotum not protrude in an angular shape	**14**
11	Pronotal lateral extensions broad, with margins subtriangular	**12**
–	Pronotal lateral extensions broad, with margins rounded	***Thlasia* Germar**
12	Body large, length longer than 19 mm; style with long fine setae on inner edge	***Macrotrichia* Zhang, Sun & Dai**
–	Body medium, length longer usually 10–15 mm; style without long fine setae on inner edge	**13**
13	Lateral extensions of pronotum broad and well developed	***Tituria* Stål**
–	Lateral extensions of pronotum narrow and not well developed	***Neotituria* Kato**
14	Body small, length 6–9 mm	**15**
–	Body moderate, longer than 9 mm	**18**
15	Center of crown with a longitudinal groove	***Petalocephaloides* Kato**
–	Center of crown with a longitudinally ridged or flat	**16**
16	Center of crown flat; base of forewing A veins not raised	**17**
–	Center of crown with a longitudinally ridged; base of forewing A veins prominent	***Parapetalocephala* Kato**
17	Aedeagus longitudinally flat or slender, with ventral process	**23**
–	Aedeagus slender, without ventral process	***Arenoledra* Kuoh**
18	Body stout; style with an odontoid process on the outside of the bend near end	**19**
–	Body slender; style without odontoid process on the outside near end	**20**
19	Forewing terminal venation reticulate; aedeagus slender	***Destinoides* Cai & He**
–	Forewing terminal venation not reticulate; aedeagus longitudinally flattened	***Destinia* Nast**
20	Crown wider than the front of pronotum; pygofer posterior margin concave	***Laticorona* Cai**
–	Crown narrower than pronotum; pygofer posterior margin not concave	**21**
21	Crown broadly rounded; aedeagus slender, terminal with 2 pairs of processes	***Pachyledra* Schumacher**
–	Crown parabolic; aedeagus without process or with 1 pair of processes	**22**
22	Forewing A_1_ vein prominent	***Platycephala* Kuoh**
–	Forewing A_1_ vein not prominent	***Petalocephala* Stål**
23	Aedeagus with paired ventral processes	***Midoria* Kato**
–	Aedeagus with single ventral process	***Parathlasia* gen. nov.**

## ﻿Taxonomy

### ﻿ Parathlasia

Taxon classificationAnimaliaHemipteraCicadellidae

Li, Jiang, Li & Xing gen. nov.

D36B667D-EAF1-5E08-BD8A-A1A0859B4432

https://zoobank.org/11D3E520-25B6-4311-9FDC-D652CB159CEF

Figures 1 , 2 , 3

#### Type species.

*Parathlasiaguizhouensis* Li, Jiang, Li & Xing, sp. nov.

#### Description.

Medium-sized, 7.5–8.0 mm long (including tegmen); yellowish to sordid brown. Head (Fig. 1A, B) with crown declivous, in dorsal view nearly twice as long and five times wider than eye; median carina complete but weakly elevated, weakly concave either side of midline, with some granular protuberances; ocelli (Fig. 1A) submarginal and close to posterior margin, closer to midline than corresponding eyes. Face (Fig. 1C) including eyes shorter than wide; frontoclypeus flattened. Pronotum slightly wider than head with anterior margin slightly convex, lateral margins oblique, slightly divergent posteriorly. Metanotum (Fig. 1A) two-thirds length of pronotum with distinct transverse depression. Forewing (Fig. 1A, B, D) with apical margin strongly oblique, three subapical cells, inner subapical open, middle subapical closed, extra apical cells present; appendix very narrow. Hind leg as in Fig. 1F, H.

**Figure 2.**
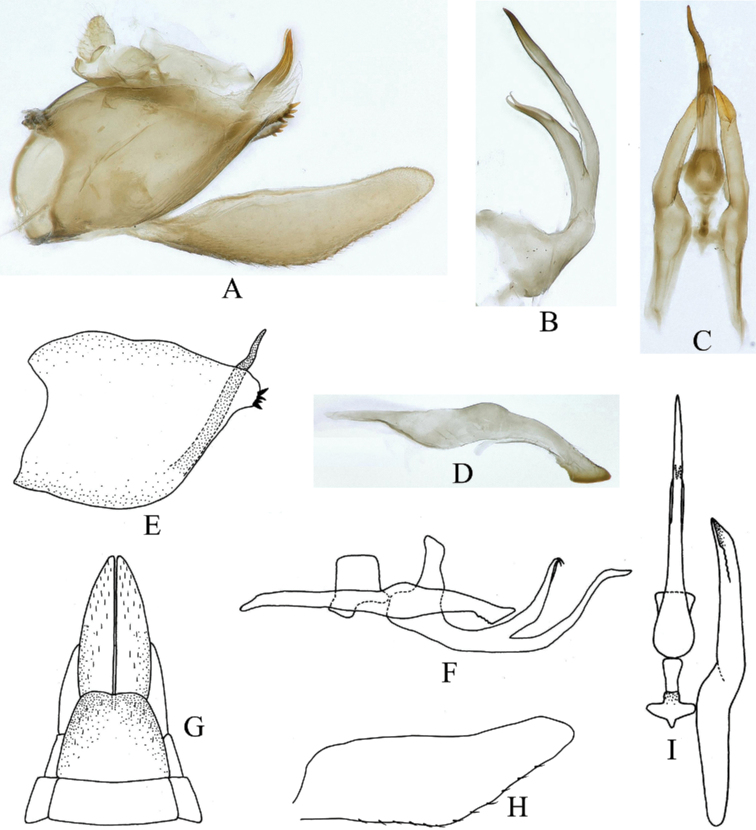
Male genitalia of *Parathlasiaguizhouensis* sp. nov. **A** genital capsule, lateral view **B** aedeagus, lateral view **C** aedeagus, connective and style, ventral view **D** right style, lateral view **E** pygofer, lateral view **F** aedeagus, connective and style, lateral view **G** apex of abdomen, ventral view **H** subgenital plate **I** aedeagus, connective and style, ventral view.

**Male** pygofer (Fig. 2A, E) with long ventrocaudal process; with some small stout setae subapically. Xth segment very short. Subgenital plates (Fig. 2A, H) fused basally, elongate, inner margin with short spine-like setae. Aedeagus (Fig. 2B, C, F, I) with shaft somewhat elongate, tubular, curved dorsally with a ventral medial process, gonopore apical on ventral surface; basal apodeme distinct. Style (Fig. 2C, D, F, I) elongate, apophysis curved ventrally, apex truncate. Connective (Fig. 2C, F, I) T-shaped.

**Female** unknown.

#### Distribution.

**Figure 3.**
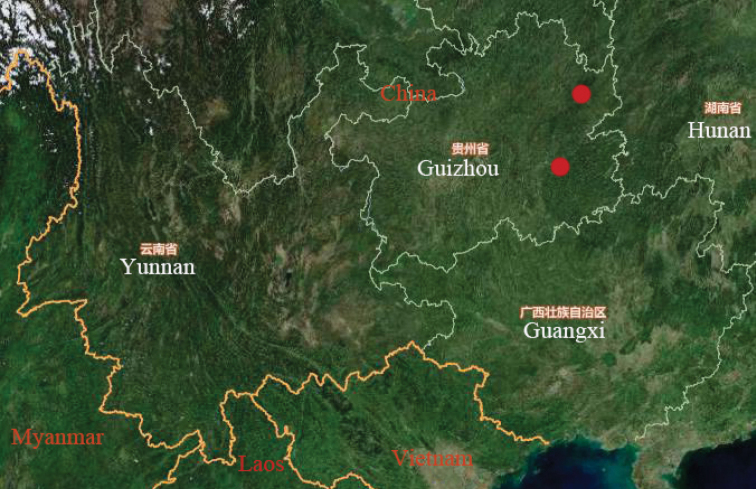
Distribution of *Parathlasiaguizhouensis* sp. nov.

China (Guizhou) (Fig. 3).

#### Etymology.

The name of the new genus refers to the similarity of the genus to *Thlasia* Germar externally.

#### Remarks.

The new genus is similar in appearance to *Thlasia* Germar, *Midoria* Kato and *Yelahanka* Viraktamath, Webb & Yeshwanth in its relatively small size with a short head and with similar extra apical forewing veins but lacking accessory cross veins. In addition, the oblique forewing apex in *Parathlasia* is also found in some species of *Yelahanka* (see Viraktamath et al. 2021) and long ventrocaudal pygofer process is found also in some species of *Thlasia* (see Zhang et al. 2004). It differs from these and other Ledrinae in having the aedeagus with a single ventral medial process (Fig. 2B, F); *Midoria* has paired ventral processes on the aedeagus (Li and Li 2010, 2011).

The new genus also appears closely related to *Parapetalocephala* Kato. The main difference between *Parathlasia* and *Parapetalocephala* are the forewing veins which in the later genus are prominent (see Jones and Deitz 2009).

### ﻿ Parathlasia guizhouensis

Taxon classificationAnimaliaHemipteraCicadellidae

Li, Jiang, Li & Xing sp. nov.

A5EE70E9-4F73-5D07-A7B6-5A425C97CAE9

https://zoobank.org/9443BF3B-7115-49C6-8F7D-D387C3B90636

Figures 1 , 2 , 3

#### Description.

Head (Fig. 1A) yellowish brown, base of crown with some darker brown marking, ocelli reddish brown. Thorax sordid brown; forewings (Fig. 1A, B, C, D) yellowish hyaline apically margined with brown.

Crown flat, more or less horizontal, surface punctate with median short ridge on posterior margin, about 0.4 times as long as wide between eyes. Ocelli not prominent, closer to each other than to adjacent eye. Pronotum shallowly foveate on either side of median line in anterior half, posterior half slightly gibbous, anterior margin slightly convex, posterior margin medially concave, lateral margin somewhat straight, about 1.85 times as long medially as crown. Mesonotum shorter than pronotum. Forewing claval region densely punctate, apical margin obliquely truncate.

Pygofer anterior margin deeply bilobed, posterior margin slightly sinuate, in lateral view about 1.2 times as long as height, ventro-cauadal process long, extending beyond dorsal pygofer margin, with some conical protrusions at end of ventral margin. Subgenital plate widest in mid-region tapering both anteriorly and posteriorly, apex acutely angled. Style broad in middle region, tapering forward and backward, apophysis curved ventrally with axe shaped apex. Aedeagal shaft (Fig. 2B, C, F, I) bifurcate apically; ventral processes elongate, curved dorsally, longer than shaft. Other male genitalia characteristics as in Figs 1 and 2.

The characteristics of female are unknown.

#### Measurement.

Length (including tegmen): ♂, 7.5–8.0 mm.

#### Type material.

***Holotype***: ♂, China: Guizhou, Fanjingshan, Huguosi, 29 May 2002, coll. Li Zizhong (QFNU). ***Paratypes***: 6♂♂, same data as holotype; 2♂♂, same data as holotype except 29 July 2001, coll. Yang Maofa (GUGC); 1♂, China: Guizhou, Leigonghan, Lianhuaping, 2 June 2005, coll. Li Zizhong and Zhang Bin (GUGC) (see Fig. 3 for geographic distributions of new species).

#### Host plant.

Unknown.

#### Etymology.

The species name is derived from the type locality.

## Supplementary Material

XML Treatment for Parathlasia

XML Treatment for Parathlasia guizhouensis
